# Supplementary material for: Feasibility of wireless continuous monitoring of vital signs without using alarms on a general surgical ward: A mixed methods study
Source: PLoS One. 2022 Mar 14;17(3):e0265435. doi: 10.1371/journal.pone.0265435 (PMC8947816; doi:10.1371/journal.pone.0265435)
Supplement: S1 Table — (PDF) [file pone.0265435.s001.pdf]

S4 Table: Shapiro-Wilk test results for normality

Variable name	Shapiro Wilk test results	
	statistic	p-value
**Patient characteristics**		
Age in years (median, IQR)	.944	.011*
Body Mass Index (kg/m^2) (median, IQR)	.954	.032*
**Healthcare professionals' characteristics (n=46)**		
Age (median, IQR)	.824	.000*
Work experience (median, IQR)	.803	.000*
**USE-questionnaire**		
Usefulness (α = .906)	.986	.855
It helps me be more effective.	.926	.006*
It helps me be more productive.	.931	.009*
It is useful.	.890	.000*
It gives me more control over the activities in my work.	.927	.007*
It makes the things I want to accomplish easier to get done.	.933	.007*
It saves me time when I use it.	.945	.011*
It meets my needs.	.928	.030*
It does everything I would expect it to do.	.943	.025*
Ease of use (α = .921)	.964	.169
It is easy to use	.884	.000*
It is simple to use	.863	.000*
It is user friendly	.856	.000*
It requires the fewest steps possible to accomplish what I want to do with it	.902	.001*
It is flexible	.832	.000*
Using it is effortless	.894	.001*
I can use it without written instructions	.919	.004*
I don't notice any inconsistencies as I use it	.889	.000*
Both occasional and regular users would like it	.889	.000*
I can recover from mistakes quickly and easily	.867	.000*
I can use it successfully every time	.877	.000*
Ease of learning (α = .842)	.968	.243
I learned to use it quickly.	.913	.002*
I easily remember how to use it.	.913	.002*
It is easy to learn to use it.	.869	.000*
I quickly became skillful with it.	.903	.001*
Satisfaction (α = .917)	.981	,655
I am satisfied with it.	.909	.002*
I would recommend it to a friend.	.919	.003*
It is fun to use.	.908	.001*
It works the way I want it to work.	.911	.002*
It is wonderful.	.921	.004*
I feel I need to have it.	.918	.003*
It is pleasant to use.	.913	.002*
**Fidelity of the monitoring system**		
Median monitoring time (median, IQR)	.751	.000*
Total measurements	.751	.000*
D-EWS scores median (IQR)	.802	.000*
D-EWS > 3 median (IQR)	.581	.000*
System notifications median (n, %)	.598	.000*
**Patient acceptability**		
Comfortable	.566	.000*
Feeling safe	.878	.000*
More involved in own health	.894	.001*
More access to healthcare professionals	.895	.001*

Recommendation for clinical use	.665	.000*
Recommendation for home use	.766	.000*
**Clinical outcomes**		
Length of stay (days)	.753	.000*
