# Supplementary material for: Feasibility of wireless continuous monitoring of vital signs without using alarms on a general surgical ward: A mixed methods study
Source: PLoS One. 2022 Mar 14;17(3):e0265435. doi: 10.1371/journal.pone.0265435 (PMC8947816; doi:10.1371/journal.pone.0265435)
Supplement: S1 Appendix — (PDF) [file pone.0265435.s002.pdf]

S1 Appendix: Thresholds EWS scores of the continuous monitoring system

	EWS score					
**Score**	**2**	**1**	**0**	**1**	**2**	**3**
**Heart rate**	**<40**	**40-50**	**51-100**	**101-110**	**111-130**	**>130**
**Respiratory rate**	**<9**		**9-14**	**15-20**	**21-30**	**>30**
