# Supplementary material for: Feasibility of wireless continuous monitoring of vital signs without using alarms on a general surgical ward: A mixed methods study
Source: PLoS One. 2022 Mar 14;17(3):e0265435. doi: 10.1371/journal.pone.0265435 (PMC8947816; doi:10.1371/journal.pone.0265435)
Supplement: S2 Appendix — (PDF) [file pone.0265435.s003.pdf]

**S2 Appendix: Questionnaire patients**

The questions below are about your experience wearing the "smart patch" to monitor your heart rate and breathing during your admittance on the ward.

You should answer the statements below on a scale of 1 to 5. 1= "strongly disagree" and 5 = strongly agree.

| | **1** | **2** | **3** | **4** | **5** |
|---|---|---|---|---|---|
| I found the patch comfortable | 0 | 0 | 0 | 0 | 0 |
| I felt safer because of the patch | 0 | 0 | 0 | 0 | 0 |
| The smart patch makes me more concerned with my own health. | 0 | 0 | 0 | 0 | 0 |
| The patch increased my access to care and contact with healthcare providers (nurses and doctors) | 0 | 0 | 0 | 0 | 0 |
| I would like to wear the patch again in the hospital next time. | 0 | 0 | 0 | 0 | 0 |
| I would also like to wear the patch at home next time after an operation. | 0 | 0 | 0 | 0 | 0 |
