# Supplementary material for: Feasibility of wireless continuous monitoring of vital signs without using alarms on a general surgical ward: A mixed methods study
Source: PLoS One. 2022 Mar 14;17(3):e0265435. doi: 10.1371/journal.pone.0265435 (PMC8947816; doi:10.1371/journal.pone.0265435)

S3 Appendix: Topics focus groups

1 General introduction and purpose of focus group

2 How did you feel about working with continuous monitoring in general?

3 We see a score of 4.4 out of 7 for usability and usefulness.

Do you recognize this? Can you explain this score?

4 We see a score of 4.7 out of 7 for ease of use.

Do you recognize this? Can you explain this score?

5 We see a score of 5.3 out of 7 for ease of learning.

Do you recognize this? Can you explain this score?

6 We see a score of 4.8 out of 7 for satisfaction.

Do you recognize this? Can you explain this score?

1. What is the value to you of active alarms during continuous monitoring?

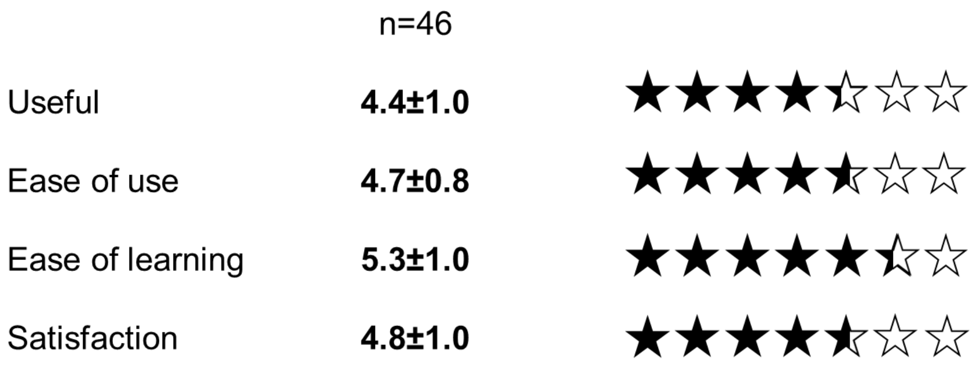

Supplement: S3 Appendix — (PDF) [file pone.0265435.s004.pdf]
