# Supplementary material for: Feasibility of wireless continuous monitoring of vital signs without using alarms on a general surgical ward: A mixed methods study
Source: PLoS One. 2022 Mar 14;17(3):e0265435. doi: 10.1371/journal.pone.0265435 (PMC8947816; doi:10.1371/journal.pone.0265435)
Supplement: S4 Appendix — (PDF) [file pone.0265435.s005.pdf]

**S5 Appendix: Distribution of answers on the USE questionnaire**

| | 1 | 2 | 3 | 4 | 5 | 6 | 7 |
|---|---|---|---|---|---|---|---|
| It helps me be more effective. | 0 (0) | 1 (2.2) | 10 (21.7) | 14 (30.4) | 12 (26.1) | 6 (13.0) | 3 (6.5) |
| It helps me be more productive. | 1 (2.2) | 5 (10.9) | 9 (19.6) | 17 (37.0) | 10 (21.7) | 4 (8.7) | 0 (0) |
| It is useful. | 0 (0) | 0 (0) | 1 (2.2) | 7 (15.2) | 11 (23.9) | 19 (41.3) | 8 (17.4) |
| It gives me more control over the activities in my work. | 0 (0) | 5 (10.9) | 9 (19.6) | 13 (28.7) | 7 (15.2) | 10 (21.7) | 2 (4.4) |
| It makes the things I want to accomplish easier to get done. | 0 (0) | 2 (4.4) | 11 (23.9) | 13 (28.7) | 12 (26.1) | 6 (13.0) | 2 (4.4) |
| It saves me time when I use it. | 1 (2.2) | 5 (10.9) | 13 (28.7) | 13 (28.7) | 8 (17.4) | 4 (8.7) | 2 (4.4) |
| It meets my needs. | 1 (2.2) | 1 (2.2) | 5 (10.9) | 16 (34.8) | 15 (32.6) | 6 (13.0) | 2 (4.4) |
| It does everything I would expect it to do. | 1 (2.2) | 3 (6.5) | 9 (19.6) | 11 (23.9) | 13 (28.7) | 8 (17.4) | 1 (2.2) |
| | | | | | | | |
| It is easy to use | 0 (0) | 0 (0) | 1 (2.2) | 7 (15.2) | 16 (34.8) | 19 (41.3) | 3 (6.5) |
| It is simple to use | 0 (0) | 0 (0) | 0 (0) | 8 (17.4) | 16 (34.8) | 19 (41.3) | 3 (6.5) |
| It is user friendly | 0 (0) | 1 (2.2) | 1 (2.2) | 5 (10.9) | 16 (34.8) | 20 (43.5) | 3 (6.5) |
| It requires the fewest steps possible to accomplish what I want to do with it | 0 (0) | 1 (2.2) | 4 (8.7) | 17 (37.0) | 10 (21.7) | 13 (28.7) | 1 (2.2) |
| It is flexible | 1 (2.2) | 0 (0) | 0 (0) | 15 (32.6) | 13 (28.7) | 16 (34.8) | 1 (2.2) |
| Using it is effortless | 2 (4.4) | 1 (2.2) | 3 (6.5) | 15 (32.6) | 13 (28.7) | 11 (23.9) | 1 (2.2) |
| I can use it without written instructions | 2 (4.4) | 8 (17.4) | 16 (34.8) | 3 (6.5) | 11 (23.9) | 4 (8.7) | 2 (4.4) |
| I don't notice any inconsistencies as I use it | 0 (0) | 0 (0) | 11 (23.9) | 16 (34.8) | 14 (30.4) | 4 (8.7) | 1 (2.2) |
| Both occasional and regular users would like it | 1 (2.2) | 1 (2.2) | 4 (8.7) | 9 (19.6) | 18 (39.1) | 12 (26.1) | 1 (2.2) |
| I can recover from mistakes quickly and easily | 1 (2.2) | 1 (2.2) | 5 (10.9) | 24 (52.2) | 10 (21.7) | 5 (10.9) | 0 (0) |
| I can use it successfully every time | 0 (0) | 2 (4.4) | 3 (6.5) | 13 (28.7) | 16 (34.8) | 12 (26.1) | 0 (0) |
| | | | | | | | |
| I learned to use it quickly. | 0 (0) | 0 (0) | 2 (4.4) | 8 (17.4) | 17 (37.0) | 13 (28.7) | 6 (13.0) |
| I easily remember how to use it. | 0 (0) | 0 (0) | 4 (8.7) | 7 (15.2) | 16 (34.8) | 12 (26.1) | 7 (15.2) |
| It is easy to learn to use it. | 0 (0) | 0 (0) | 2 (4.4) | 3 (6.5) | 16 (34.8) | 20 (43.5) | 5 (10.9) |
| I quickly became skillful with it. | 0 (0) | 0 (0) | 2 (4.4) | 10 (21.7) | 12 (26.1) | 17 (37.0) | 5 (10.9) |
| | | | | | | | |
| I am satisfied with it. | 0 (0) | 1 (2.2) | 1 (2.2) | 11 (23.9) | 18 (39.1) | 12 (26.1) | 3 (6.5) |
| I would recommend it to a friend. | 0 (0) | 0 (0) | 5 (10.9) | 10 (21.7) | 16 (34.8) | 11 (23.9) | 4 (8.7) |
| It is fun to use. | 0 (0) | 2 (4.4) | 4 (8.7) | 6 (13.0) | 12 (26.1) | 13 (28.3) | 9 (19.6) |
| It works the way I want it to work. | 1 (2.2) | 0 (0) | 11 (23.9) | 9 (19.6) | 13 (28.7) | 11 (23.9) | 1 (2.2) |
| It is wonderful. | 1 (2.2) | 2 (4.4) | 4 (8.7) | 15 (32.6) | 15 (32.6) | 8 (17.4) | 1 (2.2) |
| I feel I need to have it. | 0 (0) | 3 (6.5) | 11 (23.9) | 16 (34.8) | 11 (23.9) | 5 (10.9) | 0 (0) |
| It is pleasant to use. | 0 (0) | 0 (0) | 3 (6.5) | 13 (28.7) | 17 (37.0) | 10 (21.7) | 3 (6.5) |

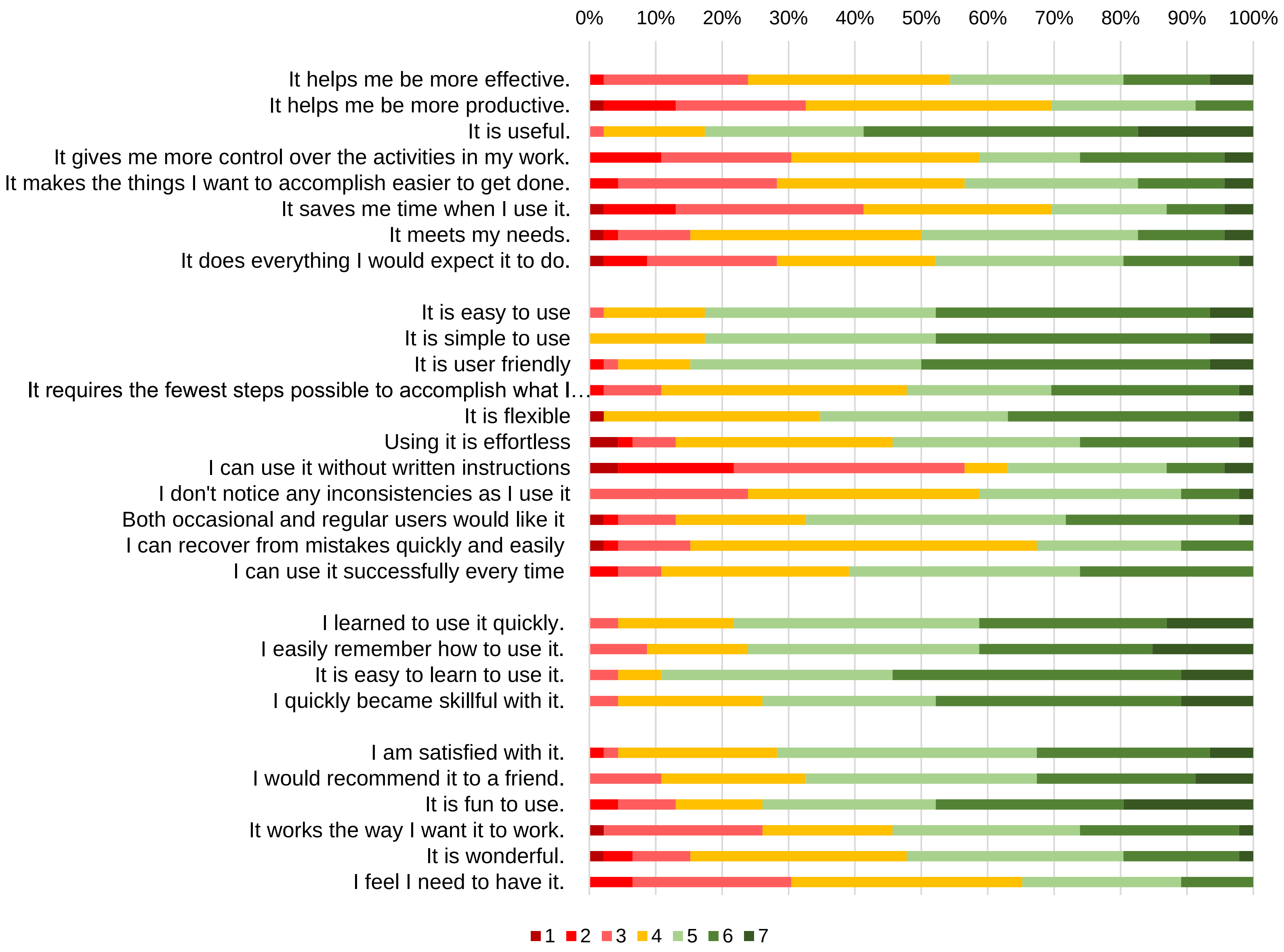
0%
10%
20%
30%
40%
50%
60%
70%
80%
90%
100%
It helps me be more effective.
It helps me be more productive.
It is useful.
It gives me more control over the activities in my work.
It makes the things I want to accomplish easier to get done.
It saves me time when I use it.
It meets my needs.
It does everything I would expect it to do.
It is easy to use
It is simple to use
It is user friendly
It requires the fewest steps possible to accomplish what I…
It is flexible
Using it is effortless
I can use it without written instructions
I don't notice any inconsistencies as I use it
Both occasional and regular users would like it
I can recover from mistakes quickly and easily
I can use it successfully every time
I learned to use it quickly.
I easily remember how to use it.
It is easy to learn to use it.
I quickly became skillful with it.
I am satisfied with it.
I would recommend it to a friend.
It is fun to use.
It works the way I want it to work.
It is wonderful.
I feel I need to have it.
1
2
3
4
5
6
7
