# Supplementary material for: Feasibility of wireless continuous monitoring of vital signs without using alarms on a general surgical ward: A mixed methods study
Source: PLoS One. 2022 Mar 14;17(3):e0265435. doi: 10.1371/journal.pone.0265435 (PMC8947816; doi:10.1371/journal.pone.0265435)
Supplement: Attachment — Submitted filename: Response to Reviewers_1.docx [file pone.0265435.s006.docx]

**Reviewers' comments

Reviewer #1: Dear editor,**
*I’ve reviewed the manuscript "Feasibility of wireless continuous monitoring of vital signs without using alarms on a general surgical ward: a mixed methods study". I would like to congratulate the authors for providing an interesting and well planned designed explanatory sequential mixed method study.

However, I would like to share my points of critique with you:

Major concerns:*

1. *For the evaluation of the "Usefulness, Satisfaction, and Ease of use" questionnaire (7-Likert scale), means and standard deviation were used. This is not statistically permissible for in particular for Likert items. Likert-items often present statistical skewness and therefore non-parametric descriptive statistics must be used here (median, range). As sum scores within the individual sub-categories (usefullness, satisfaction, ease of learning, ease of use), means including standard deviation are possible. I recommend re-analyses of the Likert items and on the basis of this a re-evaluation of the results. Moreover, results of Shapiro-Wilk test are missing.*

Thank you for addressing this issue. We do agree that Likert-items often present statistical skewness and therefore non-parametric descriptive statistics are more applicable. Therefore, we had already analyzed this with the Shapiro-Wilk of all items and constructs in the previous version of our manuscript. This resulted in non-parametric statistics for the individual items and parametric statistics for the constructs. This was not sufficiently clear in the table; therefore, we have further clarified this for every statement (Page 14; Table 3). Besides, we have also added the results of the Shapiro-Wilk tests in the Supporting information file for all continuous variables (S4 Table: Test results of Shapiro-Wilk tests).

2. *In line 233 continuous monitoring are described as useful with a mean score of 4.4 (standard deviation +/- 1.0). A score around 4 is described as "neutral". I recommend an adjustment of the interpretation of the results regarding usefulness. In addition, add a table (supplement) with an exact itemization of the Likert scale points 1-7. Within aggregated scores information for the reader get possibly lost.*

Thank you for pointing this out. We do agree the interpretation of the score 4.4 was incorrect. Therefore, we have changed this in the abstract and throughout the text. (Page 2, Line 48-49 & Page 11, Line 277-280)

*Although insight into vital sign trends allowed faster anticipation and action upon changed patient status, but professionals still were neutral about usefulness (4.4±1.0).* (Page 2, Line 48-49).

*Overall, healthcare professionals considered continuous monitoring as easy to use (4.7 ± 0.8), easy to learn (5.3 ± 1.0) and were satisfied with it (4.8 ± 1.0) but were neutral about its usefulness (4.4 ± 1.0) (Table 3).* (Page 11, Line 277-280)

We have also added a separate table with the exact itemization results of the Likert scale points in the Supporting information (Supporting information S5 Table: Distribution of answers on the USE questionnaire)

3. *The discussion is in its current form too superficial. I believe there are many aspects that can be discussed.
   For example a little more discussion on why we need these wireless continuous monitoring on a non-ICU ward, why a non-ICU without continuous monitoring should implement continuous monitoring systems and a comparison of advantages and disadvantages of this wireless system (without alarms). The discussion would really benefit.*

Thank you this comment. We do agree some aspects were discussed superficially. Therefore, we reassessed our discussion section and revised several aspects to add depth to the discussion. For instance discussing the need for alarms on the ward and assessing vital sign trends by nurses regularly (Page 21, Line 467 to Page 22, Line 518)

*Minor concerns*

4. *Line 42: Amount of professionals are missing.*

We have added this in the abstract.

*Professionals (N=46) considered continuous monitoring satisfying (4.8±1.0 on a 1-7 Likert-scale).* (Page 2, Line 42)

5. *Line 43: Range of Likert scale is missing.*

We have added this in the abstract.

*Professionals (N=46) considered continuous monitoring satisfying (4.8±1.0 on a 1-7 Likert-scale).* (Page 2, Line 42)

6. *Line 116/117: An explanation why 45 professionals were chosen to provide sufficient data? (power calculation / study reference?)*

Thank you for addressing this issue. We have revised this section about the sample size to provide an explanation in why 45 professionals were sufficient. (Page 8, Line 185)

*Sample size*

*Considering a 66% response rate in our previous study[20] in the same population, we considered a sample size of at least 45 professionals (70% response) as sufficient to determine acceptability resulting in an acceptable margin of error of 8% with a 95% Confidence Interval based up on the total population of 63 nurses.[30] We chose a pragmatical sample size of at least 45 professionals (70% of all nurses on the ward) to provide sufficient data to determine acceptability by professionals on the ward over three month period. Also, two focus groups of about 6-7 nurses each was expected sufficient to capture all views on acceptability.[31] During the pre-defined implementation period of three months aAll relevant patients were approached for participation during the pre-defined implementation period of three months, adding up to a total of 65 patients who could be included in the study.*

7. *Line 91/120: 4 month period or 3 month period?*

The total study period was 3,5 months including the preparation of the professionals by education and conducting the focus groups. The total period of working with continuous vital sign monitoring was 3 months. However, to prevent misinterpretations we have changed this into 3 months for both lines. (Page 5, Line 117)

8. *Line 116/229/table2/table3: 45 or 46 professionals? Please correct the discrepancy.*

The correct amount of professionals was 46. We therefore have checked and changed this in the manuscript.

9. *Line 166: For completeness add answers of 7-point Likert scale.*

We have added this in this sentence. (Page 9; Line 206)

*on the beliefs about the continuous monitoring system measured on a 7-point Likert scale (1=strongly disagree; 4=neutral; 7= strongly agree).*

10. *Line 118/184/235: Focus group of about 6-7 nurses are mentioned in "sample size". Whereas in "qualitative data" two focus groups with four professionals and in "acceptability ny healthcare professionals 9 nurses are described. Please correct the discrepancy.*

By the means of a sample size justification; we aimed to include 6-7 nurses per focus group. Therewith we chose for at least four nurses per group to gain sufficient discussion during the focus group. Eventually, nine nurses participated over two groups. We have clarified this in the text and discussed this in the limitations-section. (Page 9, Line 227 & Page 19, :Line)

*Two semi-structured focus groups consisting of at least a minimum of four convenience-sampled professionals each were conducted in a secluded room on the ward in the last week of the study after two random shifts.* (Page 9, Line 227)

*Fourth, we only included nine nurses in the focus groups, so data saturation may not haves been fully reached in the qualitative assessment part of this study. However, given the homogeneity of the group of nurses and low level of complexity of the topic this may not be an issue.[49]* (Page 23, Line 534)

11. *Line 184/185: For completeness add themes of topic list of focus groups.*

We have addressed this in the Supplementary files but did not referred to it. We have added the reference to it. (Page 7, Line 185)

12. *Line 203: Please add reference after mentioned stages.*

We have added the reference after mentioning the stages. (Page 8, Line 204)

13. *Line 205/206: For completeness add who conducted second, third, fourth and fifth stage.*

We have clarified this in the text. (Page 7, Line 207)

14. *Line 118/235: For completeness add recruitment information for focus group. Special criteria for including in the focus group? Random allocation?*

We have adapted this in the text and hope it made the criteria more clear. (Page 7, Line 185)

*convenience-sampled professionals each were conducted in a secluded room on the ward in the last week of the study after two random shifts.* (Page 9, Line 228)

15. *Line 233/234/table3: Please add score range.*

We have clarified this in the text. (Page 8, Line 207-208)

16. *Table 3: Descriptions within the brackets are missing. (standard deviation? percentage?)*

We have clarified this in the table per item. (Page 14)

17. *Line 346: For more clarity please add Clavien-Dindo classification for I and II complications.*

We have clarified this in the text.

*Patients were admitted for a median time of 5 days (IQR4-7) and developed a total of 21 complications of which 18 were Clavien-Dindo class I and II complications, and 3 were class 3, 4 or 5, as shown in Table 1.* (Page 18; Line 398)

Reviewer #2: Dear authors,

Thank you for the opportunity to review the manuscript, “Feasibility of wireless continuous monitoring of vital signs without using alarms on a general surgical ward: a mixed methods study in which you "

This is an up-to-date evaluation modern wearable devices for vital sign monitoring, which have recently become available for patients. Their use without alarms aims to improve alarm fatigue and inefficiencies at the workplace, which is an issue that should definitely be addressed again and again to improve working conditions ant the patient’s outcome.

This is, in principle, a well-structured / logically structured study on the basis of an acceptable data pool. However, prior a possible publication, I would like to share my thoughts on the manuscript:

18. *Since the continuous vital sign monitoring are a completely new concept on many general wards, the study is lacking a control group. You mainly addressed the opinion of healthcare professionals on the system, but the main question, whether there is a clear advantage of using continuous monitoring of vital signs for patients is not answered. It would be desirable to compare your study collective to a reference group, where “normal” monitoring is performed. Due to the lacking control group, it is not clear whether postoperative complications or deviations in vital signs requiring intervention be noticed earlier and therefore prevent complications, reduce mortality, etc*.

In our study we aimed to determine feasibility, in terms of acceptability and fidelity, of continuous monitoring on the ward without using an alarm system. We defined acceptability of the professionals as the primary outcome because this construct is the most important factor on feasibility of this innovation. Therefore, we applied a mixed-methods design to fully explore the acceptability and enrich the data of the questionnaire.

It was not our intention to determine advantage as in effectiveness of continuous monitoring of vital signs for patients. Adding a control group would not help in answering our research question about feasibility. Further, demonstrating effectiveness of earlier detection would be more relevant after feasibility has been demonstrated as also proposed by several implementation models, such as the Medical Research Council Framework for the development and evaluation of complex interventions.

- Craig, P., Dieppe, P., Macintyre, S., Michie, S., Nazareth, I. and Petticrew, M. (2008) Developing and evaluating complex interventions: the new Medical Research Council guidance. BMJ, 337.979-983.
- O’Cathain A, Croot L, Duncan E, et al. Guidance on how to develop complex interventions to improve health and healthcare. BMJ Open2019;9:e029954.

19. *Please insert the hospital name in line 92/93*

We have added the hospital name.

*An explanatory sequential mixed methods design was used to determine feasibility over a 4-month period (July-October 2020) on a 24-bed surgical ward in Isala, aof large tertiary teaching hospital in the Netherlands.* (Page 5, Line 108)

20. *In line 109 you state that biosensor-patch was attached postoperatively, however I would recommend giving the short description of the system prior to those statements in the methods part.*

We have moved the procedures section after explaining the intervention in the methods section. (Page 7, Line 175)

21. *You state that HR and RR were exclusively measured. How accurate can these vital parameters be monitored with wearable devices compared to e.g. ECG? Since you performed the study on a general ward vital signs such as blood pressure are of utmost importance as well - did you measure those parameters additionally?*

We described the accuracy of the wearable devices briefly in the introduction section of our manuscript. (Page 4, Line 71-73). We do agree other vital signs parameters are important besides HR an RR, therefore we implemented the continuous monitoring systems as a supplement to the daily intermittent manual measurement of the MEWS in this study. We clarified this in the methods section and discussed this more extensively in the discussion section. (Page 7, Line 149-150 & Page 23, Line 528-530 and 544)

22. *How was the time frame of every four hours for the routinely assessment of the vital signs trend determined?*

During a whole day of 24 hours there are three nursing shifts of 8 hours each. During the study preparations, we defined the work process of continuous vital sign monitoring in consultation with the nurses, we decided to assess the trend twice per shift (each 4 hours). We have clarified this in the text (Page 6, Line 159).

23. *With a continuous monitoring and assessment of vital sign trends a faster anticipation and action upon changed patient status may be feasible, but how can acute deviations of vitals signs which require intervention be managed?*

Thank you for this comment which is in line with the comment of the previous reviewer. We have addressed this issue in the discussion section. (Page 21, Line 468-501)

24. *Table 2: work experience “(years)” is missing.*

We have added this in the Table. (Page 13, Line 301)

25. *It would be desirable to provide a brief “outlook” on necessary changes/improvements. Throughout the manuscript, the authors list a few examples with regards to this (e.g. limited digital tools for interpretation of vital signs). Furthermore, a statement regarding a possible use of these systems on ICUs could be added.*
    We have assessed the discussion section and added an implications section in the discussion where we clustered the discussion of necessary changes in continuous vital sign monitoring interventions and suggest topics for further research. (Page 23, Line 539-549).

Further, the Philips Biosensor BX100 and Intellivue Guardian Solution software system used in the study are developed for clinical use in nursing wards and not in high care departments because it is not a patient surveillance system. We have added this in the methods section.

*The continuous monitoring system is Conformité Européene–(CE) marked and was developed as a continuous monitoring tool for general wards and not for high care units.* (Page 7, Line 148-150)
